# Metabolic Engineering Strategies for Enhanced Polyhydroxyalkanoate (PHA) Production in *Cupriavidus necator*

**DOI:** 10.3390/polym17152104

**Published:** 2025-07-31

**Authors:** Wim Hectors, Tom Delmulle, Wim K. Soetaert

**Affiliations:** Centre for Industrial Biotechnology and Biocatalysis (InBio.be), Department of Biotechnology, Faculty of Bioscience Engineering, Ghent University, Coupure Links 653, 9000 Ghent, Belgium

**Keywords:** *Cupriavidus necator*, polyhydroxyalkanoates, PHB, metabolic engineering PHA copolymers

## Abstract

The environmental burden of conventional plastics has sparked interest in sustainable alternatives such as polyhydroxyalkanoates (PHAs). However, despite ample research in bioprocess development and the use of inexpensive waste streams, production costs remain a barrier to widespread commercialization. Complementary to this, genetic engineering offers another avenue for improved productivity. *Cupriavidus necator* stands out as a model host for PHA production due to its substrate flexibility, high intracellular polymer accumulation, and tractability to genetic modification. This review delves into metabolic engineering strategies that have been developed to enhance the production of poly(3-hydroxybutyrate) (PHB) and related copolymers in *C. necator*. Strategies include the optimization of central carbon flux, redox and cofactor balancing, adaptation to oxygen-limiting conditions, and fine-tuning of granule-associated protein expression and the regulatory network. This is followed by outlining engineered pathways improving the synthesis of PHB copolymers, PHBV, PHBHHx, and other emerging variants, emphasizing genetic modifications enabling biosynthesis based on unrelated single-carbon sources. Among these, enzyme engineering strategies and the establishment of novel artificial pathways are widely discussed. In particular, this review offers a comprehensive overview of promising engineering strategies, serving as a resource for future strain development and positioning *C. necator* as a valuable microbial chassis for biopolymer production at an industrial scale.

## 1. Introduction

As a society, our widespread dependence on plastic materials is undeniable and is a direct result of their immense utility. These versatile materials come in many shapes and sizes, showing varying degrees of durability, density, weight, and elasticity [1,2]. Their wide range of possible properties is determined by the polymer backbone and sidechains that make up their hydrophobic chemical structure, but an aspect shared by most plastics is their synthetic or semi-synthetic origin, hailing from their petroleum-based production. While fossil fuels are widely regarded as an essential resource, the substantial environmental downsides brought about by their use in the plastic industry are a well-documented cause of concern. Conventional plastics are estimated to emit 2 billion metric tons CO_2_ equivalents per year, accounting for 4% of global greenhouse gas emissions [3,4]. In addition, most plastics of petrochemical origin are notoriously recalcitrant to biodegradation, causing them to amass in various ecosystems. Coastal and marine ecosystems are among the many that show rapidly progressing abundances of microplastics, with toxic consequences for biota, affecting species diversity and harming ecosystem health [5,6,7].

Despite these downsides, global production is not slowing down. Over 400 million tons of plastics were produced in 2022 worldwide, of which 80.3% were fossil-fuel based [8]. Additionally, post-consumer challenges remain, as large shares of these materials still end up being incinerated or in landfills. Although incineration has the added benefit of recovering some of the energy stored in the material, both greenhouse gases and harmful toxins are released in the process [9,10]. Recycling tackles a sizeable part of the pollution problem by reducing waste and conserving resources, for example, the conversion of plastic waste to fuel oils by pyrolysis, but issues such as a lack of steady supply, sorting difficulty, a lack of suitable catalysts, and higher economic costs render recycling technologies insufficient to fully resolve the issues accompanying this industry [11,12].

**Figure 1:**
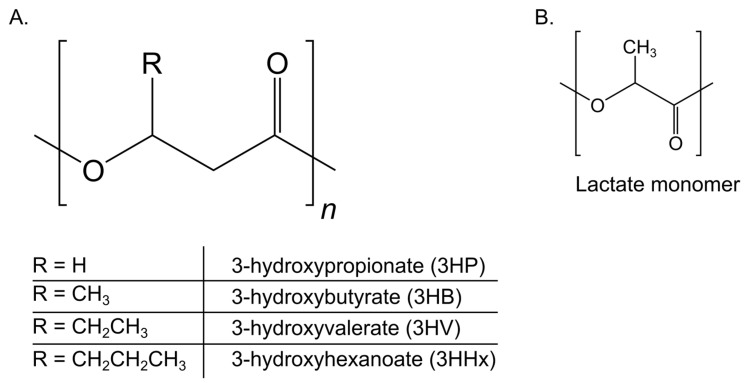
Chemical structure of polyhydroxyalkanoates (PHAs) and common monomers. (**A**) General PHA backbone with variable R-groups corresponding to key monomers: 3-hydroxypropionate (3HP), 3-hydroxybutyrate (3HB), 3-hydroxyvalerate (3HV), and 3-hydroxyhexanoate (3HHx). (**B**) Structure of the lactate (LA) monomer, found in poly(3-hydroxybutyrate-co-lactate) (P(3HB-co-LA)) copolymers. These monomers contribute to the physicochemical diversity of PHA-based biopolymers.

While research and development concerning the end-of-life destinations of plastics remains part of the solution, other viable alternatives have to be explored. Driven by a general momentum toward a bio-based economy, ‘green’ materials produced from renewable resources represent a bottom-up approach that addresses waste and pollution prevention [13,14]. Keeping emissions and waste generation in mind, plastic materials that are both bio-based and biodegradable represent an opportunity to optimize environmental benefits, as these dual characteristics ensure that the material not only reduces dependence on fossil carbon but also addresses end-of-life issues through microbial degradation. Matching these criteria are polyhydroxyalkanoates (PHAs), which were first observed in bacteria as early as 1888. Since then, research interest has steadily expanded, leading to initial industrial interest and the first patents by the 1960s, intensifying further with the rise of the focus on a bioeconomy, a timeline explored more extensively in a dedicated review by Palmeiro-Sánchez et al. (2022) [15]. These aliphatic polyesters are produced by a wide variety of microorganisms, including strains from the genera *Pseudomonas*, *Cupriavidus*, and *Bacillus* [16,17,18,19]. PHAs are accumulated intracellularly by microorganisms as a carbon and energy reserve, while they can also function to remediate external stress factors, such as nutrient limitation, salinity, UV radiation, temperature, or osmotic stress. Some strains have been reported to accumulate PHA inside granules to concentrations reaching more than 80% of the cell dry weight (CDW), indicating its potential as a viable fermentative product [20,21,22]. PHAs can be classified into three different classes based on the number of carbon atoms in their monomeric units. Short-chain-length PHAs (scl-PHAs) are made up of C_3_ to C_5_ monomeric units, medium-chain-length PHAs (mcl-PHAs) contain C_6_ to C_14_ monomeric units, and long-chain-length PHA (lcl-PHA) monomers contain more than 14 carbon atoms. Each of these classes is characterized by its well-documented and unique thermal and mechanical properties, determining its application potential. Moreover, their end-use properties can be further modified by small changes in their chemical structures or by the development of heteropolymers to further expand possible applications. This structural diversity is illustrated in Figure 1, which shows the general PHA backbone and the side chains of several key monomers found in industrially relevant copolymers. Owing to their non-toxicity, biocompatibility, and biodegradability, several of these PHAs have already received regulatory approval for applications in food contact and biomedical contexts [23]. To find an in-depth assessment of potential applications and the properties these biopolymers exhibit, as well as challenges regarding industrial production, the reader is encouraged to see more specialized reviews [23,24,25,26]. Mcl-PHAs and scl-PHAs are the most extensively researched classes, of which *Pseudomonas putida* and *Cupriavidus necator* function as their respective model production hosts [27,28]. In particular, *C. necator*, notable for its native production of homopolymer polyhydroxybutyrate (PHB), possesses multiple characteristics that make it an ideal cell factory for the production of PHAs.

*C. necator* is a Gram-negative soil bacterium that has been a topic of biopolymer research for decades, formerly denominated *Hydrogenomonas eutropha*, *Alcaligenes eutropha*, *Wautersia eutropha*, and *Ralstonia eutropha* before being designated its current name [29,30]. Its many names found in the literature are rooted in the various environments this bacterium has been sampled from, which allude to *C. necator*’s ability to metabolize a wide variety of substrates [31]. This versatility in usable substrates translates into a promising microbial chassis for PHA production compatible with the needs of multiple industries, as *C. necator* presents itself as an opportunity for upcycling a variety of waste streams [32]. However, commercialization and large-scale industrialization are lagging, as PHAs are substantially more expensive than the petroleum-based plastics they are meant to replace. Currently, the cost of PHAs is at least three times as high as that of polypropylene and polyethylene [33,34,35,36]. Large-scale production is deemed insufficiently cost-effective compared to their petroleum-based counterparts, as a direct result of PHAs’ high production costs. To bring down costs, multiple strategies for PHA production have been researched, such as the choice of substrate, substrate pretreatment, and downstream processing methods, feeding optimization strategies, and various forms of nutrient limitations [20,32,34,35]. However, these strategies mainly focus on process improvements, while optimizing the organism itself holds potential that can be harnessed through metabolic engineering and can act complementarily to the research mentioned prior [37,38,39,40,41,42,43,44,45,46,47,48]. However, key challenges in bioengineering PHA production remain, including limited dynamic control over carbon fluxes and difficulties in tailoring polymer composition with precision. Moreover, engineered strains often exhibit trade-offs between biomass formation and PHA accumulation, complicating efforts to maximize overall productivity. By optimizing metabolic pathways, expanding substrate utilization, and reducing byproduct formation, genetic modifications can aid in the improvement of productivity and further reduce costs. Such advancements could make PHA production more commercially viable and competitive, positioning these biopolymers as practical alternatives to conventional plastics. To highlight advancements made in this field and outline the most promising strategies, this review provides an in-depth understanding of *C. necator*’s native PHB production pathways and the targeted metabolic engineering strategies to increase both homo- and copolymer PHA productivity. By providing a comprehensive discussion on the impact of key genetic modifications in PHA biosynthesis in various contexts, this work updates the framework for future strain engineering. It emphasizes how metabolic engineering strategies, ranging from cofactor balancing and regulatory tuning to central carbon rewiring, enable *C. necator* to perform under diverse cultivation conditions and support high-yield PHA production.

## 2. PHB Metabolism in *Cupriavidus necator*

**Figure 2:**
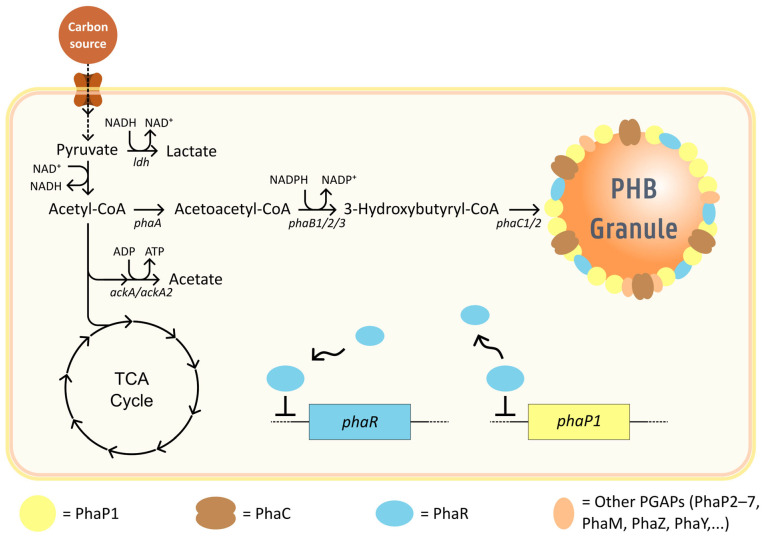
PHB metabolism in *C. necator*. Carbon is converted to pyruvate and acetyl-CoA. In nutrient-limiting conditions, acetyl-CoA is funneled to PHB formation instead of the TCA cycle. 3-hydroxybutyrate monomers are incorporated into the nascent polymer chain. The granule is surrounded by a proteinaceous layer formed by enzymes participating in PHB granule homeostasis. PHA synthase enzymes enable the polymer chain to grow, while PhaP molecules stabilize the granule, which can be expressed due to PhaR binding upstream of *phaP1*. PhaR dissociates as the granule surface saturates, its cytosolic concentration increasing and binding upstream of its own gene, autoregulating itself. Black arrows in the metabolic pathways indicate native enzymatic reactions. Dashed arrows indicate more than one reaction.

As stated before, *C. necator* is known to accumulate PHB, one of the most studied PHAs. It typically has a molecular weight related to its relatively large genome. The metabolic flexibility that *C. necator* displays regarding substrate utilization means that there are many routes that can lead to the formation of intracellular PHB granules, yet they all converge at acetyl-CoA, making this a significant point of interest for metabolic engineering. A prerequisite for PHB biosynthesis is an excess of carbon coupled with nitrogen or phosphorus limitation. Build-up of acetyl-CoA and NAD(P)H inhibits key TCA cycle enzymes, redirecting acetyl-CoA toward PHB synthesis [49]. It can also be elicited by oxygen limitation, providing an alternative sink for excess reducing equivalents [50]. Biosynthesis of PHB is predominantly driven by the constitutively expressed *phaCAB* operon, which encodes the key enzymes acetyl-CoA acetyltransferase (*phaA*), acetoacetyl-CoA reductase (*phaB1*), and PHA synthase (*phaC1*) (Figure 2) [51,52,53]. In the event of starvation, acetyl-CoA that is normally funneled to the tricarboxylic acid (TCA) cycle is instead condensed with a second acetyl-CoA molecule by the activity of *phaA*-encoded acetyl-CoA acetyltransferase (also known as β-ketothiolase for its thiolase activity) to form acetoacetyl-CoA [54]. Acetoacetyl-CoA is then converted by acetoacetyl-CoA reductase to the final precursor, (*R*)-3-hydroxybutyryl-CoA (3HB-CoA). Three functional acetoacetyl-CoA reductase enzymes have been identified in *C. necator*, encoded by *phaB1*, *phaB2*, and *phaB3*. Complementation studies have shown that PhaB1 is the main contributor to native PHB biosynthesis, which stems from its strong constitutive expression and enzymatic activity. Conversely, *phaB2* is shown to be uninvolved, as its deletion results in no phenotypic changes. However, PHB production can be partially restored in a triple reductase deletion strain when *phaB2* is integrated at the *phaB1* locus, proving PhaB2 to be a functional protein [55]. Although transcriptomics data shows upregulation of *phaB2* under oxygen-limiting conditions, the lack of beneficial effects on PHB biosynthesis further supports its limited physiological relevance [56]. Gene expression of *phaB3* is seemingly nutrient-dependent, contributing to PHB production during growth on fructose but remaining inactive during growth in rich media or on plant oils. Despite this regulation, *phaB3* largely compensates for *phaB1* disruptions, as seen in mutants lacking the latter [55]. The last step of the pathway is catalyzed by PHA synthase (PhaC), which polymerizes the monomer 3-HB-CoA via a stepwise thiol-ester mechanism, releasing the CoA molecules in the process. Among the four recognized classes of PHA synthases, the class I PHA synthase of *C. necator* is the most thoroughly studied and typically forms scl-homopolymers of PHB [57]. Similar to PhaB, more than one gene exists that encodes for PhaC, namely *phaC1* and *phaC2*. In this case, *phaC1* is the only gene contributing to PHB biosynthesis. Functional analyses have shown that *phaC2*, despite increased transcription during oxygen limitation, cannot realize PHB synthesis in the absence of *phaC1* [53,56].

PhaC occurs as a soluble protein located in the cytoplasm in cells where PHB accumulation conditions are not met. When metabolically unbalanced conditions trigger PHB granule formation, PhaC is incorporated into the nascent biopolymer granules and dimerizes to form active synthases (Figure 2) [57,58,59]. This binding to the granule is aided by its catalytic mechanism, where one of the amino acids in the active site forms a covalent bond to the growing polymer chain [57]. Thus, the PHB molecule acts as an anchor, binding the enzyme to the granule as polymerization proceeds. Anchoring of PhaC plays a crucial role in granule propagation. The resulting spatial arrangement positions the catalytic site close to the elongating polymer chain and monomer pool, facilitating granule growth [60,61].

Aside from PHA synthase, additional proteins are associated with the surface of the granular PHB structures that aid in PHB homeostasis and the granule’s formation and stabilization (Figure 2). Alongside PhaC, these gene products are referred to as PHB granule-associated proteins (PGAPs) and include regulator proteins (PhaR), phasins (PhaP1–7) that aid in the formation and stabilization of the nascent polymer granules, PHB depolymerases and oligomer hydrolases (PhaZ/Ys), which play a role in PHB homeostasis by mobilizing stored carbon from the granules, making it accessible for cell growth and maintenance, and a sixth type of PGAP called PhaM that plays a role in localization and is responsible for granule distribution during cell division [62,63,64,65,66]. While these proteins are not involved in direct conversions of precursor molecules into PHB, knowledge of their unique roles and importance in PHB homeostasis is essential for identifying possible targets of metabolic engineering strategies regarding PHB production.

While all phasins are known to play a role, PhaP1 has been identified as the primary phasin constituent on the surface of PHB [66,67]. Mutants lacking PhaP1 are known to accumulate PHB at a lower rate; they display leakage of PHB and only form one single large granule per cell as opposed to multiple smaller ones. The formation of a single large granule leads to a smaller surface area, contributing to the decrease in PHB synthesis, as less PHA synthase can be bound to the granule. PhaP1 also acts as competition to non-PHB-related proteins that might be incorporated through unspecified binding and would negatively affect PHB accumulation [67,68,69]. Other phasins have also been shown to bind to the granules in vivo, albeit in lower amounts, as well as displaying phasin–phasin interactions, illustrating the complexity of this system. Further studies dedicated to these phasins are required to fully understand their roles related to granule dynamics, which are believed to include roles overlapping with or supplementary to those of PhaP1, in addition to more specialized roles such as granule localization or DNA interactions [56,61,62,64,65,66,67,70]. Downstream of the *phaCAB* operon, *phaR* encodes a transcriptional regulator that functions as a repressor, binding upstream of *phaP1* and thereby preventing its transcription. Upon the onset of PHB biosynthesis, PhaR proteins bind the newly forming granule, lifting repression and enabling *phaP1* expression for proper granule formation. *C. necator* strains with a *phaR* disruption show a large number of comparatively small granules as a result of *phaP1* overexpression in the absence of its repressor, increasing stabilization of small PHB granules in the hydrophilic cytosol. Additionally, PhaR binding sites upstream of the *phaR* locus shed light on the autoregulation mechanism of *phaR*, which serves two critical functions (Figure 2). Firstly, it prevents unnecessary PhaP1 production prior to PHB biosynthesis. This is a crucial matter, as excessive PhaP1 imposes a significant metabolic burden on the cell, considering that it takes up 3–5% of the total cellular protein composition when PHB granules have fully matured. Secondly, as granule maturation progresses and the granule surface becomes saturated, cytosolic PhaR concentration increases, which is also intensified because of competitive displacement by PGAPs. As a result, the elevated cytosolic PhaR binds to the upstream regions of the *phaR* locus, effectively preventing its own overexpression. This feedback mechanism thus efficiently regulates PhaP1 levels in the cell and supports granule homeostasis, seamlessly adapting to the different stages of the nascent biopolymer granule [71,72]. Aside from PhaP1, other phasins (PhaP2–7) have been gradually identified. Just as with the former, transcriptomics shows their increased expression in PHB-permissive conditions [66,73]. However, PhaP1 expression is still dramatically higher, which might explain why the elimination of the minor phasin genes does not lead to phenotypical changes, suggesting negligible influence. It was only in mutants lacking *phaP1* that additional deletions of phasins PhaP2, PhaP3, or PhaP4 further impacted PHB accumulation [73]. In addition to nucleotide sequence similarities, PhaP3 shares the most traits with PhaP1, as its expression is also regulated by PhaR. Indeed, in ∆*phap1* mutants, PhaP3 is found at elevated levels compared to the wild type [74].

## 3. Metabolic Engineering Strategies to Enhance PHA Production

### 3.1. PHB

#### 3.1.1. Enhancing Production Through PHB Pathway Engineering

*C. necator* displays versatility in substrate utilization, meaning that many sources can be taken into consideration when developing strains for the purpose of enhanced PHB biosynthesis. Given the complexity of the metabolic network leading to biopolymer accumulation, the most straightforward approach for strain development entails targeting the *phaCAB* locus, where these metabolic crossroads converge. Early efforts targeted the overexpression of the enzymes encoded by this genomic locus [75,76,77]. Park et al. (1995) overexpressed combinations of *phaC1*, *phaAB*, and *phaCAB*. The greatest improvements were seen in strains with additional copies of *phaC1* and *phaCAB*, suggesting *phaC1* as the main driver of PHB biosynthesis. Interestingly, while the authors dismissed *phaAB* due to unchanged PHB titers, the lack of improvement was the result of increased polymer accumulation being offset by a decrease in biomass synthesis [75]. Thus, while increased β-ketothiolase and acetoacetyl-CoA reductase activity positively impacted PHB accumulation within cells, the effect also highlights the critical trade-off between polymer content and biomass accumulation, a key consideration for industrial application. A follow-up study by the same research group further validated these results. Additionally, β-ketothiolase activity was shown to strongly correlate with PHB accumulation rate, whereas PHB synthase activity showed marginal to no effect [77]. It should be noted that while most studies are conducted on the H16 strain, the positive effects of overexpression of native *phaC1* via plasmids have been tested and verified in strain PTCC 1615 as well [78]. Building on the knowledge that PhaC is a key enzyme in this pathway, subsequent genetic engineering strategies explored the development of mutant PhaC1 polymerases through random PCR mutagenesis. Several PhaC mutants were evaluated by introducing them via expression vector in the PHB-negative mutant *C. necator H16 PHB^−^4*, a strain carrying a truncated *phaC* [79,80]. Surprisingly, a number of reduced enzymatic activity mutants outperformed the wild-type enzyme in terms of polymer production and accumulation, contrary to earlier results in *E. coli* [76,81].

A more holistic approach is to obtain enhanced biopolymer production by modification of the interconnected pathways of the central carbon metabolism that share intermediates with or lead to the PHB biosynthetic pathway, optimizing carbon and energy flux in the process. For example, a mutant exhibiting lower isocitrate dehydrogenase activity accumulates more PHB when cultivated on sugars, as partial blockage of isocitrate dehydrogenase negatively affects TCA cycle activity. The resulting excess of acetyl-CoA cannot be metabolized during exponential growth and is instead funneled toward the PHB biosynthetic pathway, even leading to growth-associated production of PHB [82]. Conversely, this same defect results in substantially diminished yields when the mutant is grown on non-sugar substrates such as pyruvate due to cofactor imbalances. NADPH is normally generated by both isocitrate dehydrogenase and glucose-6-phosphate dehydrogenase during sugar metabolism [82,83]. This underlines the importance of balanced cofactor availability when engineering optimized PHB producers.

#### 3.1.2. Improving Cofactor Availability

Cofactor–PHB dynamics have been studied more thoroughly in multiple organisms, including *C. necator.* The investigated NADPH/NADP^+^ ratio plays a critical role in funneling acetyl-CoA away from the TCA cycle and toward the biosynthetic pathway of PHB, exerting a positive effect on acetoacetyl-CoA reductase activities [82,84,85,86,87,88]. Contrary to this positive influence, however, multiple-fold increases in NADPH levels depress cell growth, an observation that can be appreciated in these various studies, across a multitude of organisms. Intuitively, the ratio can be increased by feeding NADPH or inducing oxygen limitation [85,89]. The advantage of these methods is that NADPH levels can be increased only when entering the production phase, prioritizing biomass synthesis prior. Since a drastically elevated NADPH/NADP^+^ ratio has been shown to negatively affect cell growth, one could argue that metabolic changes directly acting on this ratio over the course of the entire microbial fermentation, entailing the growth and production phase, could produce less desirable results, as despite a possible increase in PHB content, the final concentration may suffer. However, moderate increases in this ratio appear to yield positive effects without the drawbacks for biomass generation [88,90]. For example, a study employing random mutagenesis to enhance PHB production in *C. necator* reported an increase in the NADPH/NADP^+^ ratio of 0.7 to 1.2 owing to increased activity of NADPH-generating enzymes involved in carbon metabolism. This shift was accompanied by elevated activity levels of PHB pathway enzymes, ultimately leading to a 55% improvement in PHB titers, from 4.9 g/L to 7.6 g/L. Crucially, the observed increase in cofactor availability and enzymatic activity did not compromise cell growth, highlighting the potential of this strategy as a balanced approach to optimizing PHB production [90]. Given this consideration, targets for metabolic engineering that are related to cofactor dynamics should be chosen with care. This reasoning is given further credibility by the results of Lee et al. (2003), who attempted to engineer a strain capable of improved PHB production by enhancing NADP+ reduction. By transforming a plasmid carrying the *gnd* gene encoding 6-phosphogluconate dehydrogenase (6PGDH) and forcing overexpression, more NADPH can be formed in the oxidative part of the pentose phosphate pathway. However, similar to other studies, biomass synthesis depreciated [88]. PHB accumulation was hampered as well, in part because of the lower biomass concentration. A notable observation is a decrease in acetyl-CoA concentration (58–64% relative to the wild-type strain), another cause for the depressed PHB biosynthesis. A more successful strategy employed was the overexpression of a gene encoding a transketolase originating from *E. coli*, *tktA*, in an attempt to improve intracellular concentrations of acetyl-CoA. Part of the non-oxidative pentose phosphate pathway, transketolase is directly involved in G3P formation, which is further siphoned toward pyruvate and acetyl-CoA conversion in the central carbon metabolism. Notably, a modest increase in 6PGDH activity was observed as well, alongside a roughly 50% increase in NADPH levels, explained by the higher transketolase activity, which acts as the rate-limiting step in the non-oxidative PP pathway, depending on cellular conditions [91]. In conjunction with the overabundant acetyl-CoA levels, the conservative increase in NADPH/NADP^+^ ratio synergistically acts on β-ketothiolase and acetoacetyl-CoA reductase, driving PHB production.

When relying on metabolic engineering to design novel strains, the intended fermentation environment and substrate should also be considered, as this drastically impacts the cell’s metabolism, as shown in multiple models and transcriptomics datasets [40,45,46]. Whilst most studies employ nitrogen limitation to induce the accumulation of PHB, oxygen-limited conditions are known to be an effective environment for accumulation as well, with known cases suggesting that the PHB production rate under these conditions outperforms that of nitrogen-limited cultures [92,93]. This is especially relevant for autotrophic fermentations, where oxygen levels have to be kept below 6.9% (*v*/*v*) to avoid explosion danger. To mitigate this risk, concentrations well below this lowest explosion limit (LEL) have been evaluated. However, reducing oxygen availability leads to retarded synthesis of cell proteins and nucleic acids, which in turn hurts the economic viability of the full process [92]. By studying the transcriptome of *C. necator* under autotrophic, oxygen-limiting conditions, Tang et al. (2020) devised a strategy to benefit from the high PHB yield while simultaneously attaining high cell biomass [94]. Transcriptomics data revealed downregulation of energy generation gene clusters under hypoxic conditions, explaining reduced cell growth. To improve oxygen utilization in such an environment, the authors expressed the enzyme *Vitreoscilla* hemoglobin (VHb) from a plasmid, optimizing expression using promoters derived from Johnson et al. (2018) [95]. This increased dry cell mass from 0.43 g/L to 0.55 g/L, while PHB content increased from 38.6% to 48.7% under oxygen limitation. The engineered strain also outperformed the wild-type strain in a high oxygen environment, reaching 0.97 g/L DCW and 32.3% PHB content. Secondly, to improve PHB biosynthesis, genes affecting pyruvate and acetyl-CoA were investigated. Transcriptomics showed downregulation of organic acid and TCA pathways, while the upregulation of *ldh*, *iclA*, and *ackA2* suggested the diversion of precursors to byproducts. Of these, only knockout of *ldh* improved PHB, with an 11% increase in final PHB content, likely due to the prevention of conversion of pyruvate to lactate. The other targets tested by the authors are more than one metabolic conversion removed from PHB precursors and likely cause no impact due to the intermediate steps being downregulated at oxygen levels of 3% [94]. It should be noted that a previous study on metabolite excretion by a PHB-negative mutant under oxygen-limiting conditions reported higher acetate excretion than lactate. However, the measure they used was the relative respiration rate rather than dissolved oxygen levels, making it unclear whether these conditions were below the LEL [96]. This result suggests that further investigation of *ackA2* or related genes under higher, yet still limiting, oxygen levels could prove interesting. Building on the knowledge from the transcriptomics study, the same group selected *ldh* and *ackA* as targets for deletion in a later study to improve the PHB productivity of their strains, although no further comparative experiments were performed, making it hard to gauge their impact [97].

As *C. necator*’s metabolism adapts to its environment, the avenue of oxygen limitation opens up new potential metabolic changes that could be implemented to improve PHB production. As with other prokaryotes, hypoxic conditions restrict the respiratory chain due to a lack of oxygen acting as the terminal electron acceptor, resulting in increased NADH/NAD^+^ ratios. NADH plays an important role in fermentative conditions, where several metabolites produced by microorganisms during fermentation act as a sink for excess NADH to avoid reductive stress [96,98]. On top of that, the cofactor acts as an inhibitor for the TCA enzyme citrate synthase. As the TCA cycle is inhibited, acetyl-CoA can no longer enter it [98,99,100,101,102]. Although only a few PHA-producing prokaryotes with an NADH-dependent acetoacetyl-CoA reductase have been described to date, these microorganisms, as well as other organisms engineered to contain this enzyme, have shown the ability to capitalize on the increased NADH/NAD^+^ ratio and elevated acetyl-CoA levels observed under oxygen-limiting conditions [100,102,103,104]. While *C. necator* is not among these organisms, recent structural studies of acetoacetyl-CoA reductase have provided insight into consensus sequences that play a part in cofactor specificity. This knowledge enabled researchers to redesign *C. necator*’s acetoacetyl-CoA reductase to prefer NADH over NADPH, while retaining metabolically relevant expression levels and sustaining necessary fluxes for efficient PHB production [105,106]. This opens a path toward improved PHB production processes in *C. necator* by combining anaerobic or oxygen-limited fermentations with the previously described metabolic and enzymatic changes. Aside from possible enhanced production, economic advantages include reduced energy input for the normally energy-intensive aeration and mechanical mixing, as well as reduced heat generation, leading to simplified cooling requirements [107].

#### 3.1.3. Engineering Metabolic Context Through Granule-Associated Proteins and Transcriptional Regulation

Beyond the previously discussed metabolic and cofactor modifications, PGAPs represent an often-overlooked aspect for improving *C. necator*’s PHB production processes. While their roles in granule formation and stabilization are well established, their strategic manipulation, which could offer further potential for enhancing PHB yield and productivity, remains underexplored, with only one study reporting overexpression of phasin genes to this end [56]. Through comparative transcriptomics, the authors deduced that phasin genes *phaP1* and *phaP2* are upregulated under low-oxygen stress conditions. As discussed earlier in this review, biomass synthesis suffers under these conditions, resulting in lower PHB titers despite cells reaching higher PHB contents. To avoid this yet benefit from enhanced PHB accumulation under nonstress conditions, the authors overexpressed *phaP1* or *phaP2* using expression plasmids and arabinose inducible promoter *P_Bad_*. Tuning of the expression level was necessary, as high induction of *phaP1* and *phaP2* negatively affected productivity, likely due to the high metabolic burden imposed, which is in line with ∆*phaR* strains, as discussed previously [71]. Once adequate expression levels were achieved, 49.8% and 42.9% PHB production improvement was obtained under nonstress conditions, from 0.74 g/L to 1.04 g/L and from 0.69 g/l to 0.96 g/L by overexpressing *phaP1* or *phaP2*, respectively, combating the usual decreased cell densities [56]. A possible explanation is that moderate overexpression of phasins promotes additional granule formation and stability, where more surface area allows greater allocation of PHA synthase to the granule, resulting in more efficient PHB production [67,108].

While the exploratory engineering involving phasins shows clear potential for improving PHB production, another critical avenue lies in the regulation of broader metabolic networks through transcriptional regulators. The regulatory network aids the control of gene expression, enabling the tuning of pathways involved in carbon flux, energy metabolism, and stress response. Recent studies have begun to uncover how targeted overexpression of transcriptional regulators can enhance *C. necator*’s capacity for PHB production under various conditions. For instance, the same study that explored phasin overexpression discovered a similar reaction for regulators RpoN and UspA, which respond to environmental stress, showing the ability to modulate metabolic priorities to support PHB biosynthesis under nonstress conditions [56]. Specifically, RpoN overexpression brought about a 77.5% increase in PHB titers under chemoautotrophic nonstress conditions and 103.1% under heterotrophic nonstress conditions. Surprisingly, despite the overexpression of RpoN boosting PHB productivity, as found by Jahn et. al. (2024), inactivation of RpoN led to increased fitness in a study investigating *C. necator*’s energy metabolism [47]. Another study took a different approach, engineering the overexpression of the CBB cycle master regulator CbbR and global transcriptional regulator RegA to improve growth in lithoautotrophic conditions to enhance CO_2_ assimilation [109]. Through synergistic overexpression, they achieved an 11% increase in biomass accumulation and a 28% increase in PHB titers.

A comprehensive list of key studies detailing improved PHB production in *C. necator* is given in Table 1, offering a clear overview of genetic backgrounds, engineering strategies, carbon sources, and the resulting gains in PHB titers and content.

### 3.2. PHBV

The scope of microbial biopolymer production expanded significantly with the discovery that microorganisms could synthesize copolymers when provided with suitable additional substrates. Poly(3-hydroxybutyrate-co-3-hydroxyvalerate) (PHBV) is such a copolymer, produced when 3-hydroxyvalerate (3HV) monomers are incorporated alongside 3-hydroxybutyrate (3HB), typically in a randomly distributed fashion. The addition of the 3HV fraction amends the physical properties of the PHA polymer, influencing its crystallinity, flexibility, elasticity, and melting temperature. Thus, modulating the molar ratio of 3HV in PHBV is of great interest and helps make it suitable for various industrial and medical applications [110,111,112]. Formation of PHBV starts with two precursors, acetyl-CoA and propionyl-CoA. The 3HB monomer fraction is formed as described in the previous section. When propionyl-CoA is available in the cytosol, either through metabolizing specific carbon sources or through propionate feeding, the β-ketothiolase enzyme encoded by *phaA* can also catalyze the condensation of propionyl-CoA and acetyl-CoA, forming 3-ketovaleryl-CoA. Acetoacetyl-CoA reductase then catalyzes the conversion of this intermediate to 3-hydroxyvaleryl-CoA, which serves as the direct precursor for 3HV monomer incorporation. PHA synthase subsequently polymerizes both (R)-3-hydroxybutyryl-CoA and (R)-3-hydroxyvaleryl-CoA into the nascent PHBV copolymer chain (Figure 2) [113,114,115]. The earliest commercial production of PHBV, historically sold as Biopol by Imperial Chemical Industries (ICI), relied on fermentations using *C. necator*. A drawback of this process was the necessity to use propionate (propionyl-CoA precursor) as an additional feedstock. The high costs of propionate cemented this process as too cost-ineffective, causing industrial production of PHBV to cease in the early 2000s [116,117]. Since then, endeavors have been undertaken to make microbial PHBV synthesis more economical by feedstock optimization, process improvements, and metabolic engineering [115,118,119], the latter of which will be discussed in the following section.

#### Enhancing Propionyl-CoA Supply Through Metabolic Engineering

A major cost-limiting factor of PHBV production was determined to be the reliance on exogenous propionate feeding, prompting researchers to explore alternative strategies for endogenous biosynthesis of propionyl-CoA from unrelated carbon sources. The potential of this strategy was quickly demonstrated with the development of several recombinant bacteria, such as *E. coli* and *Salmonella enterica* strains, capable of producing PHBV from carbon sources such as glycerol or monosaccharides [114,120,121,122]. In addition to these recombinant bacteria, proof-of-concept for PHBV production was also achieved in *C. necator* early on using unrelated single-carbon sources, including fructose, gluconate, lactate, succinate, and acetate, albeit with low molar fractions of 3HV (4–7%) [123]. Endogenous propionyl-CoA production was established by using a mutant that had reverted from an isoleucine-auxotrophic phenotype by overproducing acetolactate synthase, an enzyme involved in branched-chain amino acid biosynthesis. Increased flux through this pathway caused excess valine and isoleucine to be available for degradation, yielding sufficient intracellular propionyl-CoA for PHBV accumulation [123].

**Figure 3:**
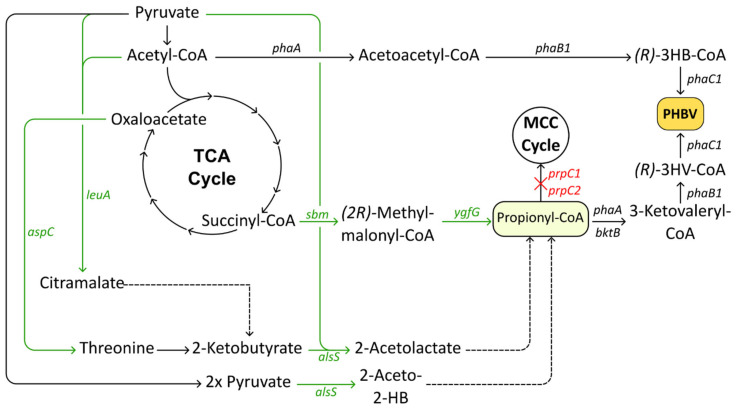
Metabolic pathways used in *C. necator* to synthesize PHBV from natively produced propionyl-CoA. Black arrows indicate native enzymatic reactions, heterologously expressed enzymes are indicated with green arrows, deletions are indicated in red and marked with ‘X’. Abbreviations: TCA cycle, tricarboxylic acid cycle; PHBV, poly(3-hydroxybutyrate-co-3-hydroxyvalerate); MCC cycle, methylcitric acid cycle; (R)-3HB-CoA, (R)-3-hydroxybutyryl-CoA; (R)-3HV-CoA, (R)-3-hydroxyvaleryl-CoA.

A notable bottleneck identified in *C. necator* for propionyl-CoA accumulation is the methylcitric acid cycle, responsible for degrading propionyl-CoA via two 2-methylcitrate synthases encoded by *prpC1* and *prpC2* [124]. These enzymes catalyze the condensation of propionyl-CoA with oxaloacetate, acting as a competing sink for propionyl-CoA, preventing 3HV incorporation. Hence, Zhang et al. (2015) deleted *prpC1* and *prpC2* in a glucose-assimilating mutant strain of *C. necator* [125]. In a shake flask setup, the resulting strain accumulated PHBV accounting for 77.4% of the CDW, with a 3HV fraction of 1.3 mol%. In addition to these knockouts, they introduced the methylmalonyl-CoA pathway by introducing a gene cluster from *E. coli*, carrying the genes *sbm*, *ygfD*, and *ygfG*. This pathway, its proficiency already demonstrated in organisms such as *H. mediterranei* and *S. enterica*, enables conversion of succinyl-CoA into methylmalonate-CoA and then into propionyl-CoA (Figure 3) [114,126]. By rewiring the central carbon metabolism to redirect carbon flux toward propionyl-CoA biosynthesis, their strain managed to achieve 68.6% PHBV of CDW, with a 3HV molar fraction of 26% and a final titer of 132.3 g/L CDW, in fed-batch cultivation conditions, a strong improvement on previously developed strains [125].

Despite the viability of the methylmalonyl-CoA pathway being demonstrated by Zhang et al. (2015), and although it is not mentioned by the authors, methylmalonate-CoA mutase encoded by *sbm* utilizes vitamin B_12_ as a cofactor [114,125,127]. Since *C. necator* lacks the complete set of genes for de novo vitamin B_12_ biosynthesis [128], the process relies on external supplementation if B_12_-dependent pathways such as methylmalonyl-CoA conversion are to be functionally expressed, hurting its economic feasibility. Thus, more recent work by Jo et al. (2023) sought to explore other pathways as alternatives. They pursued enhancing propionyl-CoA biosynthesis after investigating the feasibility of the branched-chain amino acid pathway, a method established by earlier studies [123,129]. Having first verified PHBV production following exogenous addition of isoleucine or valine, they aimed to increase flux toward biosynthesis of these amino acids by introducing a plasmid carrying the feedback-resistant gene *alsS* from *B. subtilis*, encoding acetolactate synthase, which is responsible for producing the direct precursors of both isoleucine and valine. In addition, they also overexpressed *bktB*, a native β-ketothiolase identified as the primary condensation enzyme responsible for 3-ketovaleryl-CoA production [130]. The resulting strain, which also carried a *prpC1* knockout, accumulated PHBV with a 23.6 mol% 3HV fraction but suffered from low cell mass and total PHBV content, warranting further development [129]. Alongside pyruvate, 2-oxobutyrate, or 2-ketobutyrate, is another important precursor to propionyl-CoA that is synthesized through either the citramalate or threonine pathway (Figure 3) [126,129]. By overexpressing the aforementioned pathways, 2-ketobutyrate supply and downstream target compounds can be enhanced, a strategy derived from studies in other bacterial hosts [121,131,132,133]. Expression of the heterologous genes *leuA* and *aspC*, which are involved in the citramalate and threonine pathways, respectively, was performed in *C. necator* separately, yielding the expected improvements in PHBV production. Despite both pathways having a positive effect, no attempts were made to combine both strategies in a single strain. The strain containing *leuA* significantly outperformed the strain expressing *aspC*, which the authors attribute to the citramalate pathway having a shorter route to produce 2-ketobutyrate, meaning that propionyl-CoA can be produced more efficiently than by the threonine pathway [129]. By further elimination of both *prpC1* and *prpC2*, a strain capable of producing PHBV with a 3HV fraction of up to 84.7% was achieved, albeit at the cost of both biomass and total PHA content. This was likely caused by partial blockage of the methylmalonyl-CoA pathway, which feeds directly into the TCA cycle, a phenomenon also seen in other PHBV producers [122,129]. By tuning expression levels through varying the inducer concentration or timing of induction of the heterologous genes related to PHBV production, ratios of the 3HV and 3HB fractions could be adjusted.

The success of these metabolic engineering strategies has significantly improved the feasibility of PHBV biosynthesis from cost-effective carbon sources, reducing dependence on expensive propionate supplementation. Indeed, strategies increasing the endogenous availability of propionyl-CoA by boosting precursor supply or preventing degradation have proven to enhance the bioavailability of 3HV, facilitating its incorporation into the copolymer chain. The similarities between the PHB polymer and its copolymer counterparts mean that beyond pathway modifications, previously discussed efforts focused on optimizing cofactor availability and enzyme kinetics will likely also benefit PHBV accumulation [106]. This assumption is supported by the fact that near-threefold improvements in PHBV content have been obtained under oxygen-limited conditions, following and surpassing prior successes in PHB-producing strains [94,129]. Nonetheless, observations such as unexpectedly improved cell growth under these same conditions highlight the importance of validating extrapolated strategies within the specific metabolic context of copolymer production. While the current advances mark a substantial step forward in PHBV production, further progress is needed to fine-tune flux distribution, suppress byproduct formation, and achieve yields that meet industrial demands.

### 3.3. PHBHHx

Poly(3-hydroxybutyrate-co-3-hydroxyhexanoate) (PHBHHx) is another member of the PHA family, a copolymer composed of 3-HB and 3-hydroxyhexanoate (3HHx) monomers. Unlike PHB and PHBV, which belong to the scl-PHA subgroup, PHBHHx is classified as an mcl-PHA and offers some advantages over its short-chain counterparts. Among these is improved thermal processability due to a wider processing window. While PHB melts at 170–180 °C, it undergoes rapid thermal degradation at 180–190 °C. In contrast, the incorporation of as little as 5 mol% 3HHx reduces the melting temperature of PHBHHx to below 155 °C, significantly reducing the risk of thermal degradation during processing [134]. This improvement arises from the propyl side chains of the 3HHx units, which disrupt polymer chain regularity and reduce crystallinity and melting temperature as their content increases [135]. In addition to enhanced processability, PHBHHx exhibits increased elasticity and favorable biodegradability, making it a promising candidate for a wide range of applications in tissue engineering [135,136], food packaging [137], and a variety of polymer processing and fabrication techniques [138].

Only a few bacteria have been reported to accumulate PHBHHx naturally. These include *Rhodospirillum rubrum* under phototrophic conditions [139], *Chromobacterium* sp. USM2 [140], several *Aeromonas* species including *A. caviae* FA440 [141,142,143,144,145], and a few *Pseudomonas* strains capable of incorporating 3HHx monomers [146,147,148]. To develop reliable microbial cell factories that can be fine-tuned more easily, synthetic biology approaches have been essential to enabling PHBHHx production in more tractable hosts like *C. necator.* These approaches have translated into metabolic engineering strategies that range from the establishment of new heterologous pathways to the tackling of monomer composition by targeted knockouts, which will be discussed in the following section.

#### 3.3.1. PHA Synthase

The native *phaC1* gene of *C. necator* encodes a class I PHA synthase that primarily catalyzes the polymerization of scl-monomers like 3HB and 3HV, exhibiting low specificity toward mcl-monomers [149]. More promising class I PhaC enzymes that incorporate both scl- and mcl-PHA monomers have been identified through studies examining natural producers and environmental samples by leveraging metagenomics [140,150,151,152]. Discovery of natural producer *A. caviae* led to the development of the first *C. necator* strain capable of accumulating PHBHHx by enabling the heterologous expression of *phaC_Ac_* from *A. caviae* in the PHB-negative mutant *C. necator H16 PHB^−^4*, in combination with even-carbon-number fatty acid co-feeding strategies to provide 3HHx precursors [151]. While PHB-negative *C. necator* overexpressing native *phaC1* and *phaB1* was reported to accumulate PHAs containing 3HHx monomers, incorporation levels remained minor compared to strains with a broad-specificity PHA synthase [153]. Thus, most studies rely on plasmid-based or chromosomal expression of broader specificity synthases originating from bacteria capable of naturally accumulating PHBHHx [140,151,152,154,155,156,157]. One of the more widely adopted strategies to enhance the 3HHx molar fraction has been the use of a mutated *A. caviae* PHA synthase. An approach that gained traction followed after a shift toward plant oil substrates resulted in markedly lower 3HHx monomer incorporation compared to fatty acid feeding (5% mol down from 22% mol fraction), despite advancements in PHBHHx productivity [156,157]. To address this, Kichise et al. (2002) employed directed enzyme evolution of *A*. *caviae* PHA synthase and identified two beneficial mutations comprising the single amino acid mutations N149S and D171G, which led to enhanced polyhydroxyalkanoate copolymer accumulation [158]. Both mutations were combined and evaluated in recombinant *C. necator* strains, resulting in improvements from 12.2% in the strain expressing the wild-type enzyme to 18.1% 3HHx incorporation in the double-mutant strain when grown on octanoate, and from 3.5% to 5.2% when grown on soybean oil as a single-carbon source. The N149S mutation enhanced both 3HHx incorporation and the molecular weight of the polymer, while the D171G mutation positively impacted total PHA accumulation in the cell, causing the synergistic effect to be greater than anticipated [159]. As structural insights into available PHA synthases advanced, key amino acid residues involved in the active site and substrate-binding pocket of *C. necator*’s PHA synthase were identified. Researchers built on this knowledge to construct a homology model of PhaC_Ac_NSDG using the *C. necator* enzyme as a reference, allowing structurally relevant differences at these critical locations to be mapped and potentially beneficial mutations to be found. By comparing their sequences and three-dimensional structures, three amino acid residues adjacent to the active site or the substrate entrance tunnel were found to differ; these positions likely influence substrate specificity and were identified as candidates for mutation. One of these mutations, S389T increased the 3HHx fraction from 13.1 mol% to 14.9 mol%. Threonine, being a bulkier amino acid, narrowed the substrate pocket size. Enzyme kinetics revealed a higher catalytic turnover in the mutant enzyme, possibly due to altered substrate stabilization upon entering the pocket space and accessing the catalytic site [160].

Besides PhaC_Ac_ and the mutants already discussed, various wild-type synthases from other bacteria have been introduced in *C. necator* in an attempt to improve PHBHHx production. Budde et al. (2011) tested two putative PHA synthases, PhaC1_Ra_ and PhaC2_Ra_, originating from *Rhodococcus aetherivorans*, in a *C. necator*∆*phaC1* background, with the strain expressing PhaC2_Ra_ yielding higher 3HHx content on hexanoate (11.5% vs. 18.9%) and palm oil (1.1% vs. 1.5%) [154]. Likewise, *Pseudomonas* sp. 61-3 also possesses PhaC1_Ps_ and PhaC2_Ps_ enzymes, capable of accepting 3HA units of 4 to 12 carbons. Depending on the fatty acid carbon source, 3HHx incorporation varied from 1 to 10 mol% when expressed in *C. necator H16 PHB^−^4* [161]. Similarly, Bhubalan et al. (2010) heterologously expressed PhaC_Cs_ from *Chromobacterium* sp. strain USM2 in *C. necator H16 PHB^−^4*, achieving 4 mol% of 3HHx when grown on crude palm kernel oil (CPKO) [140]. Using the same carbon source, Trakunje and coworkers only managed to produce P(3HB-*co*-3HHx) by heterologous expression of a PHA synthase originating from *Rhodococcus pyridinivorans* BSRT1-1, which was isolated from a soil sample in Thailand [162]. Finally, a class I PHA synthase mined from a mangrove soil sample through functional metagenomics was evaluated in *C. necator H16 PHB^−^4*. A 7 mol% 3HHx fraction was obtained using CPKO as the substrate, while 18 mol% was achieved through a fructose and hexanoate co-feeding strategy [152].

Together, these studies highlight that the choice of PHA synthase critically affects the 3HHx composition in PHBHHx copolymers. Substrate specificity and the ability to efficiently incorporate 3HHx monomers into the nascent copolymer chain may be caused by differences in substrate pocket and entrance tunnel structures, traits that can be altered through directed mutagenesis of key amino acid residues. Although some wild-type synthases from species like *A. caviae*, *Pseudomonas*, and *Rhodococcus* already offer broader substrate specificity compared to *C. necator*’s PHA synthase, engineering efforts continue to improve their 3HHx incorporation and polymer molecular weight. In all cases, the use of non-native PHA synthases has proven essential for overcoming the limited substrate specificity of *C. necator*’s PHA synthase, enabling the incorporation of 3HHx into the PHA copolymer chain, especially when coupled with appropriate genetic backgrounds and carbon sources.

#### 3.3.2. Engineering the β-Oxidation Pathway and Enoyl-CoA Hydratases

**Figure 4:**
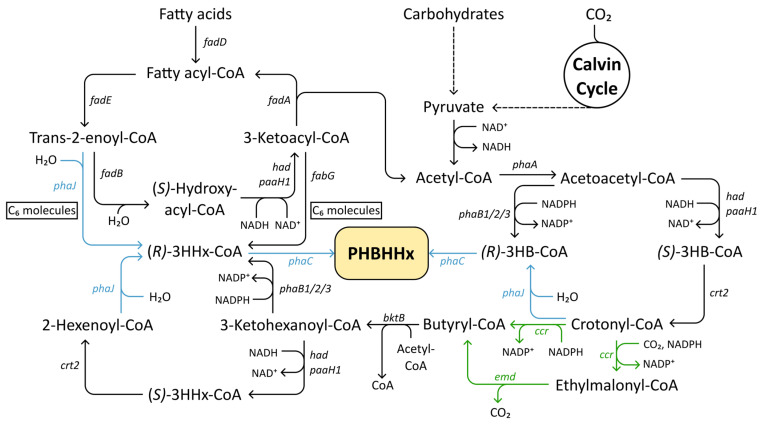
Metabolic pathways involved in PHBHHx biosynthesis. Once fatty acids have been broken down into C_6_-carbon molecules, they can enter the 3HHx pathway. Black arrows indicate native enzymatic reactions, heterologously expressed enzymes are indicated with green arrows, and genes for which both heterologous and native variants have been used are depicted with blue arrows. Abbreviations: PHBHHx, poly(3-hydroxybutyrate-co-3-hydroxyhexanoate); (S/R)-3HHx-CoA, (S/R)-3-hydroxyhexanoyl-CoA; (S/R)-3HB-CoA, (S/R)-3-hydroxybutyryl-CoA.

Many strategies exploit the β-oxidation pathway to generate medium-chain CoA precursors for PHBHHx. Through this native fatty acid degradation route, fatty acids are broken down in a cyclical manner to supply the cell with acetyl-CoA, which can be used to provide 3HB-CoA precursors. However, during β-oxidation, trans-2-enoyl-CoA molecules with a six-carbon backbone can interact with (*R*)-specific enoyl-CoA hydratases to form (*R*)-3HHx-CoA monomers for PHBHHx production instead of being further degraded (Figure 4) [163]. The enzyme responsible for this conversion, PhaJ, was first identified in *A. caviae* and generates (*R*)-3HA-CoA monomers with a chain length of C_4_–C_6_ through β-oxidation for PHBHHx formation [164]. Kawashima et al. (2012) uncovered that *C. necator* encodes three PhaJ homologs, termed PhaJ4a, PhaJ4b, and PhaJ4c. Gene disruption elucidated their roles in *C. necator*, with PhaJ4a deletion severely impacting 3HHx incorporation, whereas PhaJ4b/c did not affect the 3HHx composition of the copolymer. Notably, however, overexpression of either PhaJ4a or PhaJ4b through plasmid-borne expression or chromosomal integration in the *pha* operon outperformed the parent strain significantly in both cases, ranging from 7.2 mol% 3HHx to 8.9%, demonstrating their potential in supplying additional 3HHx-CoA monomers from 2-hexenoyl-CoA through the β-oxidation pathway. In tandem with PhaJ_Ac_ from *A. caviae*, a 10.5 mol% 3HHx composition was reached, without negatively impacting total PHA content [165]. In practice, heterologous PhaJ-encoding genes have often been introduced to boost flux toward 3HHx monomer incorporation. Chromosomal expression of both *phaC_Ac_NSDG* and *phaJ_Ac_* in *C. necator* growing on soybean oil increased 3HHx fractions from 2.6 mol% to 5.9 mol% without affecting total PHA accumulation. This was further increased to 9.9 mol% by deletion of native *phaA*, accompanied by a significant decrease in 3HB monomer and total PHA accumulation, as this limited 3HB incorporation by limiting flux to acetoacetyl-CoA and 3HB by proxy [166]. In a follow-up study, the same research group improved PhaJ_Ac_ expression by integrating it downstream of the strongly induced *phaP1* locus, resulting in a fourfold enhancement in 3HHx incorporation. Integration of a second *PhaC_Ac_NSDG* copy in the same locus further increased this to a 6.5-fold increase. Finally, by replacing the native *phaP1* with the *phaP* gene from *A. caviae*, the 3HHx fraction was increased to 17.2 mol% [167]. Modulation of PhaJ expression to control the 3HHx composition of PHBHHx is a strategy that was further pursued by Arikawa et al. (2016), who managed to produce a range of 2.8 to 10.7 mol% 3HHx-containing copolymers. Evaluating multiple constitutive gene expression cassettes revealed that stronger expression correlated with higher 3HHx compositions under the same culture conditions, showcasing that promoter strength can be used as a tool to fine-tune monomer composition in the production of PHBHHx [168]. Other examples of the use of heterologous PhaJ genes include a study by Budde et al. (2011), where *phaJ1_Pa_* originating from *P. aeruginosa* slightly outperformed PhaJ_Ac_; both improved 3HHx fractions to slightly over 22%, but PhaJ1_Pa_ conferred a slightly higher total PHA content [154]. In conjunction with *phaC_BP-M-CPF4_*, which was mined from mangrove soil samples, expression of *phaJ1_Pa_* yielded 11 mol% and 14 mol% 3HHx fractions when grown on palm oil and CPKO, respectively, up from 3 mol% and 6 mol%, respectively [169]. In more recent research, an (*R*)-enoyl-CoA hydratase from *Streptomyces* sp. strain CFMR 7 was evaluated in *C. necator*, on the same plasmid as *phaC_BP-M-CPF4_*. In this case, the copolymer contained 12 mol% and 18 mol% 3HHx fractions when grown on palm oil or CPKO, respectively, up from 4 mol% and 7 mol%, respectively [170].

In addition to modulating (*R*)-enoyl-CoA hydratase activity, metabolic engineering strategies have also been used to target other β-oxidation pathway enzymes in order to improve 3HHx monomer availability. Insomphun et al. (2014) evaluated the effects of disrupting *fadB* homologs, both singular and combinatorial knockouts, in various recombinant *C. necator* strains grown on palm oil. Disruption of *fadB1* yielded the most consistent positive result for PHBHHx production, with 3HHx accumulation increasing around 0.6–1.4 mol%, accompanied by very minimal total PHA content loss. The authors deduced that FadB1 exhibited higher affinity for short- and medium-chain-length 2-enoyl-CoA than their long-chain-length counterparts, causing the lack of this enzyme to lead to more precursor availability for (*R*)-3HHx-CoA formation [171]. There have been reports of 3-ketoacyl-acyl carrier protein reductase (FabG) conferring the ability to supply (*R*)-3HA-CoA monomers for PHA copolymer biosynthesis in recombinant *E. coli*, but heterologous expression of a *fabG* gene from *Pseudomonas* sp. 61–63 did not enhance PHBHHx in recombinant *C. necator* in any notable manner [172,173,174,175].

#### 3.3.3. Artificial C_4_–C_6_ Pathway via Crotonyl-CoA Carboxylase/Reductase and Ethylmalonyl-CoA Decarboxylase

To break away from the typical reliance on plant oils and alkanoates, an innovative approach to copolymer biosynthesis sought to establish an artificial pathway capable of building C6 monomers de novo from three acetyl-CoA molecules, bypassing β-oxidation. Foundational work by Fukui et al. (2002) pioneered this concept by expressing the *Streptomyces cinnamonensis* gene *ccr*, encoding crotonyl-CoA carboxylase/reductase (CCR), to generate butyryl-CoA from crotonyl-CoA [176]. Following native reverse β-oxidation, butyryl-CoA is elongated to form 2-hexenoyl-CoA in a stepwise manner by BktB, a β-ketothiolase with broader substrate specificity than PhaA, NADH-dependent (*S*)-3-hydroxyacyl-CoA dehydrogenase Had, and (*S*)-specific enoyl-CoA hydratase (crotonase) Crt2, which is finally converted to 3HHx monomers by PhaJ (Figure 4) [177,178]. Overexpression of *ccr_Sc_* and *phaC-J_Ac_* in *C. necator H16 PHB^−^4* led to the successful production of PHBHHx from fructose, albeit with only 1.6 mol% 3HHx incorporation [176]. Evaluation of another heterologous *ccr* gene originating from *Methylobacterium extorquens* revealed that 3HHx incorporation could be improved by increasing crotonyl-CoA carboxylase/reductase activity [175]. The low amount of C6 incorporation in the research by Fukui and coworkers can be partially explained by the significantly lower relative activity of acetoacetyl-CoA reductase toward 3-ketohexanoyl-CoA when compared to acetoacetyl-CoA, funneling precursors toward (*R*)-3HB-CoA instead. Strains lacking *phaB1* are shown to accumulate PHA copolymers with a higher fraction of 3HHx, namely 2.4–6.7 mol%. Disruption of both *phaB1* and *phaB3* increased these values to 17.8–19.2 mol% but resulted in a total of 7 wt% PHA [175,176]. Effects of *phaB1* disruption were further asserted by another group, which also showed that integration of low-activity paralog PhaB2 into the *phaCAB* operon increased PHA production compared to the *phaB1* deletion mutants. While 3HHx incorporation was lower, it was increased compared to strains retaining *phaB1* [177].

A key finding in optimizing the new artificial pathway was the newly discovered reductive carboxylase side activity of the CCR enzyme, converting crotonyl-CoA to the undesired byproduct ethylmalonyl-CoA in the presence of HCO_3_^−^/CO_2_ [179,180]. To redirect metabolic flux toward butyryl-CoA, expression of the *Mus musculus* enzyme ethylmalonyl-CoA decarboxylase was established (Figure 4). In a *C. necator* strain lacking *phaB1* and in which native *phaC1* and *phaA* were replaced by *phaC_Ac_NSDG* and *bktB*, co-expression *of ccr_Me_*, *emd*, and *phaJ4a* on a single plasmid enabled PHBHHx synthesis with a 3HHx content of approximately 22 mol%, and 38 mol% when *phaB3* was also deleted, showcasing the potential of this novel synthetic pathway [175]. Characterization and analysis of the enzymes Had and Crt2, responsible for (*S*)-3-hydroxyacyl-CoA dehydrogenase and crotonase activity, presented a novel opportunity for enforcing flux redirection to butyryl-CoA from acetoacetyl-CoA via (*S*)-3HB-CoA. Zhang et al. (2019) integrated an additional copy of the genes encoding these enzymes into the *pha* operon to further strengthen reverse β-oxidation and enhance PHBHHx biosynthesis, achieving approximately 26 mol% 3HHx in a *phaB1* deletion mutant from glucose. Furthermore, they also established robust PHBHHx production from glycerol and fructose [177]. Using the artificial C_4_–C_6_ pathway, production of PHBHHx was also achieved in *C. necator* using either sucrose or CO_2_ as sole carbon sources [181,182]. Recently, Huong et al. (2024) provided new insights into PHBHHx production under microaerobic conditions, showing that this strategy can outperform traditional nitrogen limitation in enhancing 3HHx incorporation. In an effort to elucidate the genes involved in the native reverse β-oxidation under oxygen-limited conditions, crucial roles were confirmed for *paaH1*, *had*, *bktB*, and *phaJ4a* in de novo (*R*)-3HHx-CoA biosynthesis. Disruption of these genes in glucose-grown cultures severely impacted PHBHHx production, in one case completely abolishing copolymer synthesis. Then, comparing strains missing some or all three PhaB paralogs to a parental strain with these genes intact, expression of the artificial reverse β-oxidation pathway driven by Ccr*_Me_*, Pha4Ja, and Emd*_Mm_* highlighted a synergistic effect with the native counterpart in a low-aerobic environment. While the artificial pathway alone achieved 9.8 mol% 3HHx and the native pathway only achieved 3.9 mol%, their combination resulted in copolymers containing approximately 38 mol% 3HHx [183].

### 3.4. Other PHA Copolymers

Since the discovery of *C. necator*’s ability to amass biopolymers in significant amounts, it has been widely studied for the production of both homo- and copolymers. Besides the well-characterized examples discussed previously, the search for PHAs with specialized material properties has led to the exploration of other less extensively studied yet valuable PHAs. This section will cover the metabolic engineering strategies developed to enable and optimize production for these lesser known biopolymers in *C. necator*.

#### 3.4.1. P(3HB-co-LA)

Most research pertaining to lactate-containing polyesters has been conducted in *E. coli* [184,185,186,187]. However, due to *C. necator*’s reputation as an efficient PHA producer, especially from renewable resources, it has also been tested as a host for enhanced P(3HB-co-LA) production. A patent filed by KAIST and LG Chem in 2006 describes a recombinant *C. necator* strain capable of producing P(3HB-co-LA) grown on a carbon source mixture of lactate and glucose. Incorporation of lactate into the PHA polymer was enabled by plasmid-based expression of a gene encoding a propionyl-CoA transferase derived from Clostridium propionicum. This enzyme activates lactate to (D)-lactyl-CoA, enabling incorporation into P(3HB-co-LA) [188]. Park et al. (2013) devised multiple strategies to optimize P(3HB-co-LA) production in *C. necator*, starting with the replacement of the native phaC1 gene with the Pseudomonas sp. 6–19 phaC1437 gene to mediate the low substrate specificity toward 2-hydroxyacid-CoA molecules. Additionally, while *C. necator* contains a native propionyl-CoA transferase with a broad substrate specificity, pct540 originating from Clostridium propionicum was integrated into the phaAB locus to make up for a lack of desired monomer incorporation, potentially due to the low activity of the native enzyme [189,190]. It should be noted that the research group did not evaluate the differences between strains containing the heterologous propionyl-CoA transferase and those lacking it in regard to P(3HB-co-LA) production. Finally, in this new strain, they expressed a plasmid-based ldhA gene encoding lactate dehydrogenase from *E. coli*, driving conversion of pyruvate to lactate, achieving production of P(3HB-co-LA) with a 37 mol% LA fraction from glucose as the sole carbon source. However, with a titer of 0.14 g/L PHA and 33.9% total PHA content, copolymer yield was relatively low [189]. In a follow-up study, substrate utilization of the same strain was expanded to sucrose, resulting in P(3HB-co-LA) biosynthesis with a 21.5 mol% LA fraction and a copolymer content of 19.5 wt% [191].

#### 3.4.2. P(3HB-co-3HP)

In the search for novel microbially produced copolyesters, the cultivation of *C. necator* in a nitrogen-free medium with 1,5-pentanediol, 1,7-heptanediol, or 3-hydroxypropionate (3HP) as a sole carbon source yielded the production of poly(3-hydroxybutyrate-co-3-hydroxypropionate) (P(3HB-co-3HP)), albeit with a 3HP fraction limited to 7 mol% or lower [192]. However, reliance on the addition of 3HP or related compounds into the media impedes economic viability. For example, one of the most prominent routes for microbial synthesis of P(3HB-co-3HP) is the glycerol-utilizing *pduP* pathway, which requires vitamin B12 supplementation [193,194]. To circumvent these issues, a novel synthetic route was developed in *C. necator* to supply 3HP monomers for P(3HB-co-3HP) production using structurally unrelated carbon sources. Fukui et al. (2009) established a malonyl-CoA-based pathway by expressing two genes as part of a CO_2_-fixation pathway found in *Chloroflexus aurantiacus*, through which 3HP-CoA would be formed [195]. First, acetyl-CoA is converted to malonyl-CoA by natively expressed acetyl-CoA carboxylase (ACC), which is then further converted to 3HP in a two-step reaction by the activity of heterologous malonyl-CoA reductase (MCR). Conversion of 3HP to 3HP-CoA is catalyzed by the N-terminal part of *C. aurantiacus*’ propionyl-CoA synthase, referred to as either 3HP-CoA synthetase or CoA ligase [195,196]. Native PhaC1 activity then catalyzes the condensation of both 3HB-CoA and 3HP-CoA to synthesize the desired copolymer. Using fructose as a carbon source, the authors reached a 2.1 mol% 3HP fraction, attributing relatively low incorporation to insufficient CoA ligase activity [195]. In a recent study, this same pathway was recreated using alternative genes to evaluate P(3HB-co-3HP) biosynthesis based on plant oils as the carbon source. In addition to the native *acc* gene of *C. necator*, *acc ADBC*, encoding an ACC enzyme and sourced from *E. coli*, was incorporated into the plasmid-based operon. Additionally, MCR was fragmented, based on earlier studies confirming enhanced 3HP synthesis through this method, and an evolved variant of its C-terminal was employed [197,198]. Furthermore, conversion to 3HP-CoA was instead catalyzed by a propionyl-CoA transferase mutant originating from *C. propionicum*. Molar fractions of 3HP reached approximately 32 mol% through fed-batch fermentation, reaching titers of 3.1 g/L [197].

A second artificial pathway to provide 3HP-CoA precursors, starting from exogenously supplied β-alanine, was implemented in *C. necator* based on 3HP production pathways established in other hosts [199,200,201]. This pathway was tested in a strain lacking its three methylmalonate semialdehyde dehydrogenase genes (*mmsA1-3*) to prevent 3HP consumption during copolymer biosynthesis [202]. Conversion of β-alanine to 3HP was enabled by expression of *E. coli* genes *BAPAT* and *ydfG*, encoding β-alanine pyruvate aminotransferase and an NADP-dependent 3-hydroxyacid dehydrogenase, respectively. The resulting strain acted as a proof-of-concept, yielding a copolymer with low incorporation of 1.27 mol% 3HP, consistent with previous 3HP polymer studies [195,201,203]. To improve 3HP activation, McGregor et al. (2021) evaluated multiple CoA ligation strategies by expressing either a propionyl-CoA synthetase (PrpE) or a CoA-transferase (Pct) from *C. necator*, as well as homologous enzymes from *E. coli* (PrpE and AtoAD) [201]. Out of these, only overexpression of the native Pct enzyme showed beneficial effects, improving incorporation of 3HP 10-fold. Further improvements were made by additional plasmid-based expression of either native PhaC1 or PHA synthase from *Chromobacterium* sp. USM2, yielding significantly increased 3HP molar fractions of 77–80%. By feeding different levels of β-alanine and supplementing cysteine, the copolymer’s 3HP fraction could be increased to 80–89 mol%, although total PHA accumulation suffered considerably as the 3HP molar fraction increased past 8 mol%. Moreover, the deletion of native *phaA* and *phaB1* genes, impeding 3HB-CoA formation, allowed the accumulation of approximately 91 mol% 3HP in P(3HB-co-3HP). This result was matched in a bioreactor batch cultivation, with a final titer of 1.6 g/L PHA [201].

To provide an overview of all studies detailing metabolic engineering strategies that focus on improving copolymer accumulation and the enrichment of non-3HB monomer content, a comprehensive list is presented in Table 2.

## 4. Conclusions

Over the past few decades, *C. necator* has been established as a microbial chassis for the production of both PHA homo- and copolymers, owing to its natural ability to store large quantities of PHAs. While many factors impact final yields, it is clear that metabolic engineering plays a central role in enhancing strain performance and economic viability. Despite the similarities in polymer biosynthesis, it is apparent that each PHA variant requires specific engineering strategies, from overexpression or repression of the native *phaCAB* operon constituents to the selection of PHA synthases and ketothiolases with optimal substrate specificity. This is particularly evident in the case of copolymer biosynthesis, where increasing the desired monomer fraction often comes at the cost of reduced polymer yield, highlighting a persistent trade-off that future work must address. Additionally, an important economic factor of polymer biosynthesis remains the choice of substrate, especially so for copolymers where metabolic engineering is required to decouple monomer availability from precursor feeding. To maintain relevance and scalability, future engineering efforts for polymer synthesis from unrelated, renewable carbon sources are indispensable.

Conversely, despite the diversity in monomer structures, several overarching strategies can improve total PHA accumulation across polymer types. These include central carbon metabolism rewiring, cofactor balancing, and adapting the cellular metabolism to novel fermentation strategies such as oxygen limitation. Such interventions can enhance precursor supply while minimizing byproduct formation.

In summary, rational metabolic engineering of *C. necator* enables control over polymer composition and yield, but optimizing both simultaneously remains challenging. To advance the field, future research should focus on improving monomer incorporation efficiency, minimizing biomass trade-offs, and ensuring compatibility with cost-effective, renewable, or waste-derived substrates.

## Figures and Tables

**Table 1:** Metabolic engineering strategies improving PHB production in *C. necator*. Only strategies achieving a positive impact have been depicted here. F: fermenter-level, SF: shake flask-level. (c): Information unavailable in the study but calculated based on CDW and PHB content. * Heterotrophic conditions, ** chemoautotrophic conditions.

Genetic Background	Target Genes/ Enzymes	Engineering Approach	Carbon Sources	PHB Titers (g/L)	PHB Content (%)	PHB Titer Improvement	Study
*C. necator PHB^−^4*	*phaCAB*, *phaAB*, *phaC*	Plasmid-based overexpression	Fructose	4.89 F	53.2	22–48%	[75]
*C. necator PHB^−^4*	Mutant *phaC*	Plasmid-based overexpression	Fructose	1.51 (c) SF	75.0	41%	[76]
Glucose-utilizing mutant of *C. necator H16*	-	Rewiring TCA cycle through random mutagenesis	Glucose, Fructose, Gluconate	5.52 SF, 4.59 SF, 7.27 SF	48.0, 51.0, 58.6	38.3%, 18.9%, 10.4%	[82]
*C. necator H16*	-	Cofactor balancing through random mutagenesis	Waste frying oil	7.6 SF	87.9	55%	[90]
*C. necator H16*	*tktA* from *E. coli*	Cofactor balancing, plasmid-based expression	Gluconate	Approx. 3.4 (c) SF	72.3	Approx. 60%	[88]
*C. necator H16*	VHb from *Vitreoscilla*	Increase oxygen availability, plasmid-based expression	CO_2_	0.27 SF **	48.7 **	61% **	[94]
*C. necator H16*	∆*ldh*	Gene deletion, byproduct elimination	CO_2_	0.30 SF **	41.7 **	11% **	[94]
*C. necator H16*	*phaP1*, *phaP2*, *uspA*, *rpoN*	Inducible plasmid-based overexpression	Fructose, CO_2_	0.78–1.26 SF * 0.26–0.29 SF **	30.2–47.2 *, 24.0–32.4 **	103.1% * 77.5% **	[56]

**Table 2:** Metabolic engineering strategies improving PHA copolymer production and non-3HB monomer fractions in *C. necator* F: fermenter-level, SF: Shake flask-level. (c): Information unavailable in the study but calculated based on CDW and PHB content. * Heterotrophic conditions, ** chemoautotrophic conditions.

Polymer Type	Genetic Background	Target Genes/ Enzymes	Engineering Approach	Carbon Sources	PHA Titers (g/L)	PHA Content (%)	Monomer Fraction	Study
PHBV	Isoleucine-auxotrophic revertant *C. necator H16*	Acetolactate synthase	Random mutagenesis, overexpression	Fructose, Gluconate, Acetate, Succinate, Lactate	-	47.0 SF, 35.7 SF, 29.5 SF, 21.5 SF, 43.2 SF	7%, 6%, 4%, 7%, 4%	[123]
PHBV	Glucose-utilizing mutant of *C. necator H16*	∆*prpC1C2*, *sbm-ygfD-ygfG* from *E.coli*	Gene deletion, plasmid-based expression, methylmalonyl-CoA pathway introduction	Glucose	90.76 F	68.6	26%	[125]
PHBV	*C. necator H16*∆*prpC1C2*	*bktB*, *alsS* from *B. subtilis*, *leuA* from *H. mediterranei*	Gene deletion, plasmid-based expression, citramalate and branched-chain amino acid pathway introduction	Fructose, CO_2_	0.40 (c) SF *, 0.64 (c) SF *, 1.25 (c) SF **	31.0 *, 42.5 *, 54.5 **	84.7 *%, 64.9 *%, 24.1 **%	[129]
PHBHHx	*C. necator PHB^−^4*	PhaC_Ac_	Plasmid-based overexpression, heterologous PhaC	Hexanoate, Octanoate	-	72 SF, 96 SF	28%, 15%	[151]
PHBHHx	*C. necator PHB^−^4*	PhaC_Ac_NSDG	Site directed mutagenesis of heterologous PhaC, plasmid-based expression	Octanoate, Soybean oil	2.96 (c) SF, 1.63 (c) SF	87, 71	18.1%, 5.2%	[159]
PHBHHx	*C. necator H16;* ∆*phaC*, ∆*phaZ1Z2Z6*, *P_trc_RBS-phaJ4b*	PhaC_Ac_NSDG-S389T mutant	Site-directed mutagenesis of heterologous PhaC, plasmid-based expression	Palm kernel oil	14.1 SF	83.7	14.9%	[160]
PHBHHx	*C. necator H16*	∆*phaC::* *phaC_Ac_NSDG-phaJ_Ac_*, ∆*phaA*	Gene deletion, 3HHx through β-oxidation pathway	Soybean oil	3.8 SF	79	9.9%	[166]
PHBHHx	*C. necator H16*	Δ*phaC::* *phaC_Ac_NSDG-phaJ_Ac_-phaJ4a*, ∆*phaA*	Gene deletion, 3HHx through β-oxidation pathway	Soybean oil	3.9 SF	82	10.5%	[165]
PHBHHx	*C. necator H16*	Δ*phaC::* *phaC_Ac_NSDG*, ∆*phaP1::* *phaP_Ac_-phaJ_Ac_- phaC_Ac_NSDG*	Heterologous enzyme variants, increase in gene copy number, 3HHx through β-oxidation pathway	Soybean oil	5.0 SF	79	17.2%	[167]
PHBHHx	*C. necator H16;* ∆*phaC:: phaC_Ac_NSDG*, ∆*phaZ1Z2Z6*	*::P_trc_RBS-phaJ4B*	Promoter engineering, 3HHx through β-oxidation pathway	Palm kernel oil	15.3 SF	84.0	10.7%	[168]
PHBHHx	*C. necator H16*	∆*phaC::* *phaC2_Ra_-* *phaJ1_Pa_*, ∆*phaB1-3*	Gene deletion, heterologous enzyme variants, 3HHx through β-oxidation pathway	Palm oil	0.57 (c) SF	40.4	22.44%	[154]
PHBHHx	*C. necator PHB^−^4*	*P_phaC1_-* *phaC_BP-M-CPF4_-phaA-phaJ1_Pa_*	Plasmid-based expression, heterologous enzyme variants, 3HHx through β-oxidation pathway	Palm oil, CPKO	2.5 SF, 3.0 SF	49.6, 62.6	11%, 14%	[169]
PHBHHx	*C. necator PHB^−^4*	*P_phaC1_-* *phaC_BP-M-CPF4_-phaJ_Ss_*	Plasmid-based expression, heterologous enzyme variants, 3HHx through β-oxidation pathway	Palm oil, CPKO	2.7 SF, 3.5 SF	53.5, 62.2	12%, 18%	[170]
PHBHHx	*C. necator H16; ΔphaC:: phaC_Ac_NSDG-phaJ_Ac_-phaJ4a*, ∆*phaA*	∆*fadB1*	Gene deletion, 3HHx through β-oxidation pathway	Soybean oil	34.3 F	65.7	15.7%	[171]
PHBHHx	*C. necator PHB^−^4*	*phaC_Ac_*, *phaJ_Ac_*, *ccrSc*	3HHx through *ccr* pathway, plasmid-based expression	Fructose	0.49 (c) SF	39	1.6%	[176]
PHBHHx	*C. necator H16; ΔphaC:: phaC_Ac_NSDG*, ∆*phaA::bktB*	∆*phaB1B3*, *P_phaP1_-ccr_Me_-phaJ4a-emd_Mm_*	Gene deletion, plasmid-based expression, 3HHx through *ccr-emd* pathway	Fructose	0.59 SF	41.1	37.7%	[175]
PHBHHx	*C. necator NSDG-GG*, *glucose-utilizing mutant and enhanced glycerol assimilation*	∆*phaB1::had-crt2*, *P_phaP1_-ccr_Me_-phaJ4a-emd_Mm_*	Gene deletion, increased copy numbers of reverse β-oxidation genes, 3HHx through *ccr-emd* pathway	Glucose, Fructose, Glycerol	2.1 SF, 2.1 SF, 0.5 SF	69, 75, 40	26%, 24%, 17%	[177]
P(3HB-co-LA)	Glucose-utilizing mutant of *C. necator H16*	∆*phaCAB::* *phaC1_Ps6-19_-pct_Cp_*, *ldhA* from *E. coli*	Gene deletion, heterologous enzyme variants, plasmid-based expression	Glucose	0.14 SF	33.9	37%	[189]
P(3HB-co-3HP)	*C. necator JMP134*	*mcr_Ca_*, *acs_Ca_*	Plasmid-based expression, 3HP through malonyl-CoA pathway	Fructose	0.78 (c) SF	31	2.1%	[195]
P(3HB-co-3HP)	*C. necator H16*	*Pct_Cp_*, *acc ADBC*, *N-mcr*, *C-mcr*	Plasmid-based expression, 3HP through malonyl-CoA pathway, fragmeted *mcr*	Soybean oil	3.11 F	51	32.25%	[197]
P(3HB-co-3HP)	*C. necator H16*∆*mmsA1-3*	∆*phaA*, *P_phaC_-BAPAT_Cv_-ydfG_Ec_*, *P_trp_-pct_Cn_-phaC_Cs_*	Gene deletion, plasmid-based expression, 3HP through exogenous β-alanine, heterologous enzyme variants	Gluconate and β-alanine	1.6 F	25.54	91.19%	[201]

## Abbreviations

The following abbreviations are used in this manuscript:

PHA	Polyhydroxyalkanoate
PHB	Polyhydroxybutyrate
PHBV	Poly(3-hydroxybutyrate-co-3-hydroxyvalerate)
PHBHHx	Poly(3-hydroxybutyrate-co-3-hydroxyhexanoate)
P(3HB-co-LA)	Poly(3-hydroxybutyrate-co-lactate)
P(3HB-co-3HP)	Poly(3-hydroxybutyrate-co-3-hydroxypropionate)
CDW	Cell dry weight
TCA	Tricarboxylic acid
scl-PHA	Short-chain-length polyhydroxyalkanoate
mcl-PHA	Medium-chain-length polyhydroxyalkanoate
lcl-PHA	Long-chain-length polyhydroxyalkanoate
PGAP	PHB granule-associated protein
CPKO	Crude palm kernel oil
ACC	Acetyl-CoA carboxylase
MCR	Malonyl-CoA Reductase
CoA	Coenzyme A
3HB	3-Hydroxybutyrate
3HHx	3-Hydroxyhexanoate
3HV	3-Hydroxyvalerate
LEL	Lowest explosion limit
